# Supplementary material for: A transmission electron microscope study of Néel skyrmion magnetic textures in multilayer thin film systems with large interfacial chiral interaction
Source: Sci Rep. 2018 Apr 9;8:5703. doi: 10.1038/s41598-018-23799-0 (PMC5890272; doi:10.1038/s41598-018-23799-0)
Supplement: Supplementary file 1 — Supplementary Information [file 41598_2018_23799_MOESM1_ESM.pdf]

A transmission electron microscope study of Néel skyrmion magnetic textures in multilayer thin film systems with large interfacial chiral interaction

Supplementary Information

S. McVitie[1], S. Hughes[1], K. Fallon[1], S. McFadzean[1], D. McGrouther[1], M. Krajnak[1]*, W. Legrand[2], D. Maccariello[2], S. Collin[2], K. Garcia[2], N. Reyren[2], V. Cros[2], A. Fert[2], K. Zeissler[3], C. H. Marrows[3]

In this document we provide additional information regarding:

1. Details of experimental measurements for extrapolated domain wall width from Fresnel images.
2. MuMax simulation of a domain wall in a film with DMI.
3. Proposed method to measure the chirality of the Néel domain wall from Fresnel images using simple simulations

S1. Domain wall measurement from Fresnel images

In the paper we present defocused Fresnel images of Néel walls in films with chiral texture. As this is a defocus method the width of the measured intensity associated with the walls will be wider than the actual wall width. However it is known that a simple relationship exists [1] for divergent (black) walls where the measured width, W, is linearly related to the actual wall width W_0 through the defocus Δ by the equation:

$$W = W_0 + m\Delta \qquad (1)$$

Thus a graph of the measured width W versus Δ gives a gradient m and intercept W_0. For the measurements made here the graph is show below in Fig. S1. It should be noted that the defocus values here correspond to the lens DAC values away from focus position for accuracy. The graph of measured width with varying defocus is shown in Fig. S1(a) and an example measurement from a Fresnel image in Fig. S1(b). The defocus range in Fig S1(a) corresponds to ~3-8 mm and the intercept gives a value of W_0 = 27 ± 8 nm.

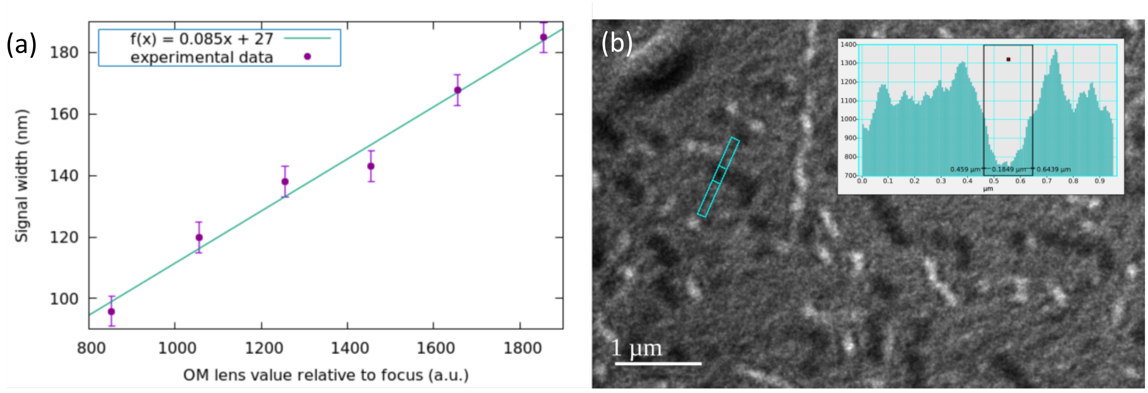

Figure S1. Domain wall measurements by Fresnel imaging. (a) Graph of measured width, in nm, for divergent domain wall as a function of lens focus. The least square fit is shown in the inset. (b) Fresnel image of domain walls with intensity taken from region indicated in blue, averaged over 15 lines. The inset shows the intensity line trace and width measurement

S2. Micromagnetic simulation of domain wall

For comparison with the experimental images a micromagnetic simulation of an isolated domain wall in a continuous film was performed using the GPU-accelerated MuMax3 software [2]. The simulation area was chosen to have dimensions of 256 nm by 256 nm in-plane with a thickness of 0.6 nm. The cell discretisation used was 1×1×0.6 nm, well below the exchange length for the material. Periodic boundary conditions were used for the in-plane directions and the material parameters used were M_s = 1.1×10^{6} A m^{-1}, A_{ex} = 16×10^{-12} Jm^{-1}, K_u = 9×10^{5} Jm^{-3} and D = 2×10^{-3} Jm^{-2}. The simulation relaxed into a Néel wall configuration as is shown in Figures S2(a-c). The spatial variation of the magnetisation components in Figures S2(a) and (c) are shown in Figure S2(d).

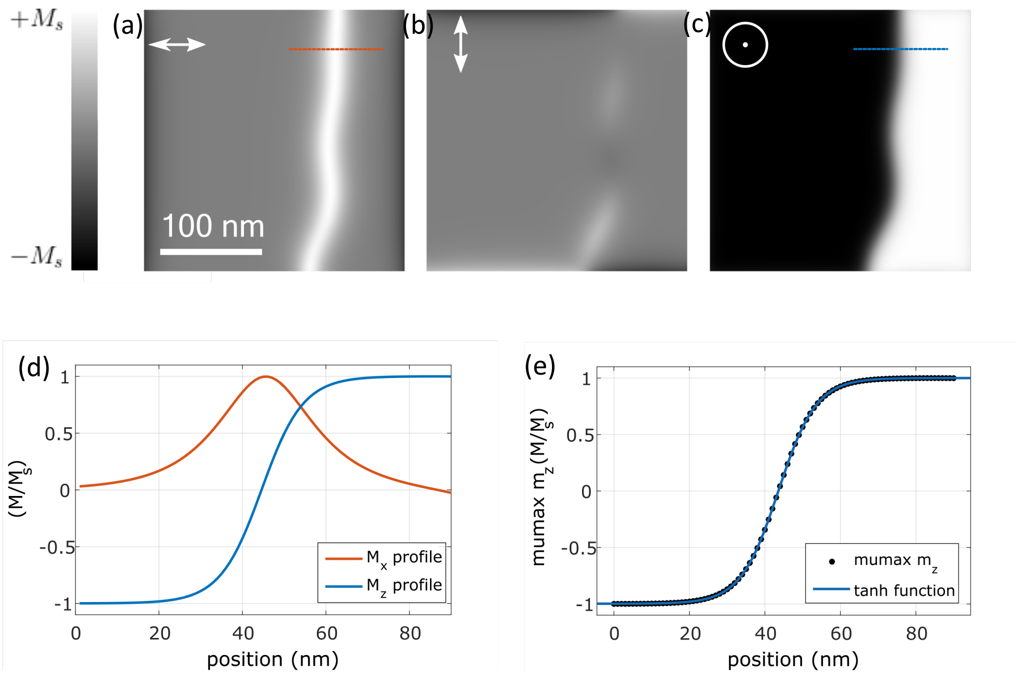

Figure S2. Micromagnetic simulation of Néel wall. (a)-(c) Magnetisation components calculated from the simulation as indicated by the symbols. (d) Profile of magnetisation for the M_x and M_z components indicated from the regions marked in red and blue in (a) and (c) respectively. (e) Comparison of M_z component with a fitted hyperbolic tangent function, ((M_z/M_s)tanh(x/A)), with fitting parameter A = 10 nm giving FWHM (from M_x component) of 27 nm.

Néel walls are commonly well represented by a trigonometric function and here the z-component of magnetisation can be represented by:

$$M_z = M_s \tanh\left(\frac{x}{A}\right) \qquad (2)$$

This is shown in Figure S2(e) which is a normalised M_z profile (plotting M_z/M_s) together with a fitted tanh function with an A parameter of 10 nm. The width of this profile from the FWHM of the M_x component is 27 nm which is excellent agreement with both the extrapolated Fresnel width and the directly measured DPC value.

S3. Proposed method to determine Néel wall chirality using Fresnel images

Lorentz TEM imaging has been used to determine that domain walls in materials with DMI are of Néel character [3,4]. However it should be noted that the argument proving that the walls are indeed Néel type arises from a lack of contrast from the divergent (e.g. M_x in Fig s2(a)) magnetisation component in the Fresnel image. This is certainly true when the electron beam is tilted with respect to an axis perpendicular to the length of the domain wall. However if the beam is tilted away from this axis it is possible that the divergent component [5] may contribute to the image contrast. This would then allow the sense of the chirality to be determined in addition to verification of the Néel character of the wall. To show how this may be possible we take the magnetisation profile from equation (2) for M_z and then the M_x component given by:

$$M_x = \pm\sqrt{M_s^2 - M_z^2} \tag{3}$$

The magnetisation components associated with this 1D model wall for a pair of domain walls are shown in Figure S3(a-c). It can be seen that the M_x component is consistent with a defined chirality of the magnetisation. Fresnel images were calculated for 200 keV electrons and a material with M_s = 1.1×10^6 A m^{-1} and a thickness of 0.6 nm. Three different configurations were considered with a beam tilt of 45° at different azimuth angles as indicated in Figures S3(d)-(f). The images were calculated for a defocus value of 0.5 mm. As expected when the tilt is perpendicular to the length of the wall the wall profiles are symmetric with the divergent (M_x) magnetisation component having no contribution to the image, Figure S3(e). However when the azimuth angle is rotated by 45° a hint of asymmetry in the wall profiles at the two different angles is visible indicated by Figures S3(d) and (f). This can be seen more prominently when taking intensity linetraces across the walls and shown in Figures S3(g-i). The divergent component contribution can be seen in these linetraces where the asymmetry at edges of the wall profile goes up/down or down/up relative to the uniform contrast in the domains. This noticeably is inverted when comparing Figures S3(g) and (i) due to the changed projection of the magnetisation within the walls onto the electron beam. Of course the contrast from the wall component is much smaller than the peak contrast that arises from the domains. However it is clear that the sense of the domain wall and hence the sign of *D* could be determined by this method.

Looking at the wall contrast from the experimental images, notably the linetrace in Figure S1(b) it can be seen that the background signal variation in the film makes detection of such an asymmetry challenging. At present we have not been able detect any asymmetry unambiguously. Furthermore the asymmetry arsing requires the magnetic structure to be uniform throughout the thickness. For some of the multilayer films imaged here recent evidence suggests that the wall structure may vary through the thickness [6]. As such any asymmetry may cancel between top and bottom layer contributions if the Néel rotation changes through the stack as suggested there. We have though demonstrated that it may be possible in principle to determine the sign of *D* in films with uniform structure throughout the thickness using Lorentz TEM.

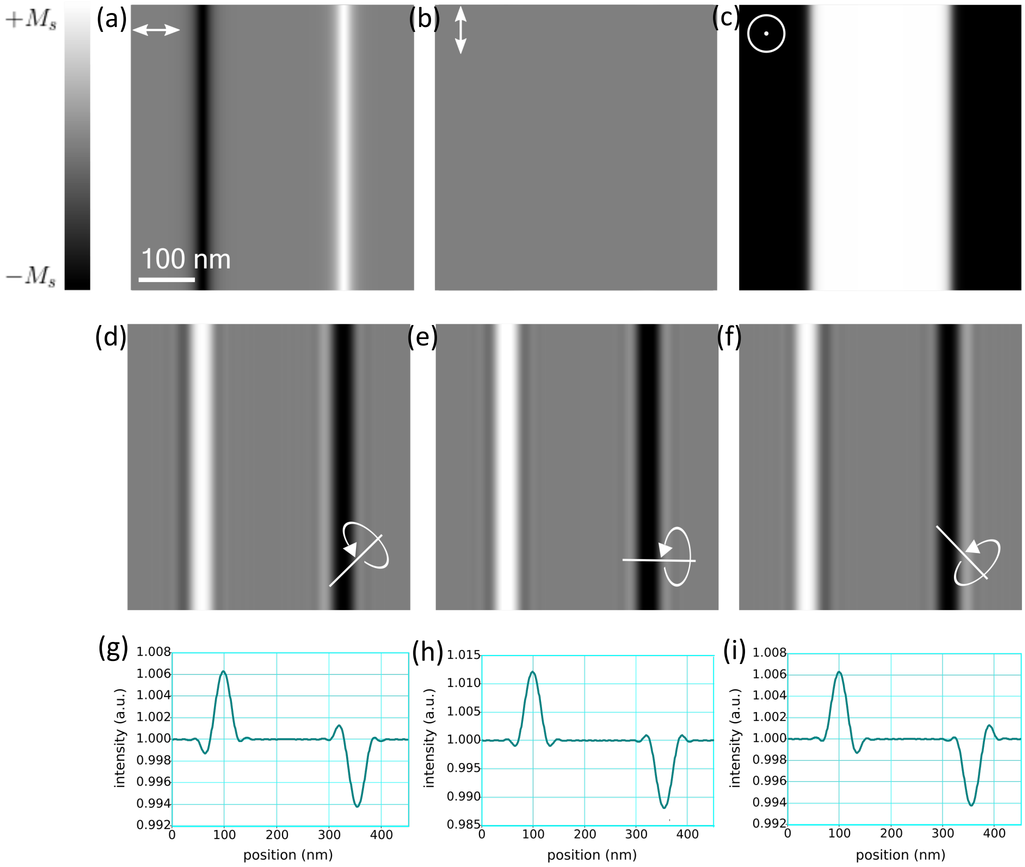

Figure S3. Proposed determination of Néel wall sense by Fresnel imaging. (a)-(c) Magnetisation components modelled on a profile ((M_z/M_s)tanh(x/A)) with parameter A = 10 nm. (d-f) Calculated Fresnel images for the electron beam tilted with respect to the sample as indicated by the lines and arrows. In each case the defocus used was 0.5 mm. (g-i) Show linteraces from the calculated images in (d-f) respectively showing asymmetry in the domain wall profiles in (g) and (i) due to the contribution from the M_x wall component.

[1] Gong, H., & Chapman, J. N. On the use of divergent wall images in the Fresnel mode of Lorentz microscopy for the measurement of the widths of very narrow domain walls. *J. Magn. Magn. Mater.* **67**, 4 (1987).

[2] Vansteenkiste, A. *et al.* The design and verification of MuMax3. *AIP Advances* **4**, 107133 (2014).

[3] Benitez, M. J. *et al.* Magnetic microscopy and topological stability of homochiral Néel domain walls in a Pt/Co/AlOx trilayer. *Nat. Commun.* **6**, 8957 (2015).

[4] Pollard, S. D. *et al.* Observation of stable Néel skyrmions in cobalt/palladium multilayers with Lorentz transmission electron microscopy. *Nat. Commun.* **8**, 14761 (2017).

[5] Mansuripur, M., J. Appl. Phys. **69**, 2455 (1991).

[6] Legrand, *et al.*, Room-temperature current-induced generation and motion of sub-100 nm skyrmions. *Nano Letters* **17**, pp. 2703-2712, (2017).
